# Localized Pneumocystis jirovecii pneumonia in a malnourished, non-HIV-infected man in the absence of any established or diagnosed immunosuppressive condition: a case report

**DOI:** 10.1186/s12890-024-03308-y

**Published:** 2024-10-07

**Authors:** Mohammad Javad Fallahi, Pariya Kouhi, Seyed Amir Sadrzadeh, Mansoureh Shokripour, Massood Hosseinzadeh

**Affiliations:** 1 Thoracic and Vascular Surgery Research Center, Shiraz University of Medical Sciences, Shiraz, Iran; 2 Department of Internal Medicine, School of Medicine, Shiraz University of Medical Sciences, Shiraz, Iran; 3 Student Research Committee, Shiraz University of Medical Sciences, Shiraz, Iran; 4 Department of Pathology, School of Medicine, Shiraz University of Medical Sciences, Shiraz, Iran

**Keywords:** Pneumocystis jirovecii pneumonia, Malnutrition, Pulmonary infection

## Abstract

**Background:**

Pneumocystis jirovecii infection is an opportunistic infection that mostly affects patients with immunosuppressive conditions like human immunodeficiency virus (HIV) infection or medications, like corticosteroids. This study reports a rare case of Pneumocystis Jiroveci infection in a relatively immunocompetent patient which presented with uncommon radiological findings.

**Case presentation:**

A 46-year-old man with a malnourished appearance and a history of opium dependence presented with dry cough, dyspnea, and weight loss to the hospital. There was no evidence of an immunocompromised condition or use of any immunosuppressive medication in the history of the patient. A lung high-resolution computed tomography (HRCT) scan revealed a crazy-paving appearance and localized infiltration. Methenamine silver staining and the histopathological findings in the transbronchial lung biopsy confirmed the diagnosis of PJP. Antibiotics and bronchodilators were administrated and the patient was discharged after 6 days of hospitalization. HIV testing and immunoglobulin levels were normal in the hospital course as well as his follow-up visits. After a 2-month follow-up, the patient was in good condition despite of mild remaining infiltration in his lung.

**Conclusions:**

PJP typically affects HIV-infected patients, but due to excessive use of immunosuppressive medications, its prevalence is increasing in non-HIV-infected patients. Malnutrition may predispose the patients to PJP, even in the absence of immunosuppressive conditions.

## Background

Pneumocystis jirovecii, previously called Pneumocystis carinii, is an atypical fungus that mostly infects immunocompromised patients, especially HIV-infected patients. It can cause pneumonitis, and without early diagnosis and treatment, it has a high rate of respiratory failure and mortality. Nonspecific respiratory and constitutional symptoms or abnormal radiological findings demand diagnostic evaluations of Pneumocystis jirovecii pneumonia (PJP) in all immunocompromised patients, including direct examination of bronchoalveolar lavage (BAL) fluid and induced sputum samples, quantitative polymerase chain reaction (PCR) and direct visualization of PJP microorganisms in lung tissue as the gold standard for diagnosis [1–3]. In this paper, a case of PJP in a non-HIV patient without any immunosuppressive conditions or medication is reported with atypical radiological findings.

## Case presentation

The patient was a 46-year-old current cigarette smoker male with opium dependence, who presented with gradual dyspnea (modified Medical Research Council (mMMC) grade 4) and dry cough that started 2 months before hospital admission. He also experienced significant unintentional weight loss for 2 years, severe anorexia, and poor exercise compliance, probably due to advanced opium addiction. There was no history of fever, or hemoptysis. Given the patient’s medical history of chronic expectoration and previous use of bronchodilator inhaler, chronic obstructive pulmonary disease (COPD) exacerbation was the primary differential diagnosis. Physical examination revealed a skinny and malnourished man with no respiratory distress at rest or any minimal activity with an oral temperature of 37.1 °C, blood pressure of 125/75 mmHg, heart rate of 100 beats/min with a regular rhythm, respiratory rate of 21/min and O2 saturation on room air measured with a pulse oximeter of 87%. On posterior chest examination, lung auscultation revealed bilateral diffuse expiratory wheezing and fine mid inspiratory crackle on the lower lobe of the right lung (below the tip of right scapula and right costophrenic angle). Anterior chest examination also revealed diffuse bilateral expiratory wheezing. Other physical examinations were unremarkable. ECG was normal without any ischemic changes or rhythm irregularity. Venous blood gas analysis showed a pH of 7.45, a partial pressure of carbon dioxide (PCO2) of 30.6 mmHg, and a serum bicarbonate (HCO3^−^) of 21.4 mEq/L. The blood tests indicated transient leukopenia (1700 cells per microliter to 4500 cells per microliter), and serum immunoglobulins (IgM, IgG, and IgA) levels were within normal limits. HIV antibody/antigen tests were also negative. The complete laboratory results are presented in Table 1:

**Table 1:** Complete labratoary data

Index	Unit	Normal range	Admission	Discharge
WBC	*10^3^/µl	4–10	1.7	4.5
			*PMN = 46% lymphocyte = 38%*,* metamyelocyte = 8%*,* monocyte = 8%*	
Hb	g/dl	12–16	12.9	12.5
MCV	fl.	80–96	85.8	86.2
PLT	*10^3^/µl	150–450	112	150
FBS	mg/dl	70–100	69	-
BUN	mg/dl	8–20	41	26
Cr	mg/dl	0.4–1.2	1.1	0.67
Na	mEq/dl	136.145	131	137
K	mEq/dl	3.5–5.5	4.5	-
Phosphorus	mg/dl	3-4.5	3.6	-
Calcium	mg/dl	8.6–10.3	9	-
AST	Units/l	< 40	46	-
ALT	Units/l	< 34	26	-
ALK	Units/l	80–306	64	-
Globulin	g/dl	2-3.5	2.9	-
Albumin	g/dl	3.5–5.2	3.7	-
Total protein	g/dl	6-7.8	6.6	-
Direct bilirubin	mg/dl	< 0.3	0.4	-
Total bilirubin	mg/dl	0.1–1.2	1	-

*Note* g = gram, dl = decileter (1 decileter = 0.1 liter), fl = femtoliter (1 femtoliter = 10^-15^ liter)

**Fig. 1:**
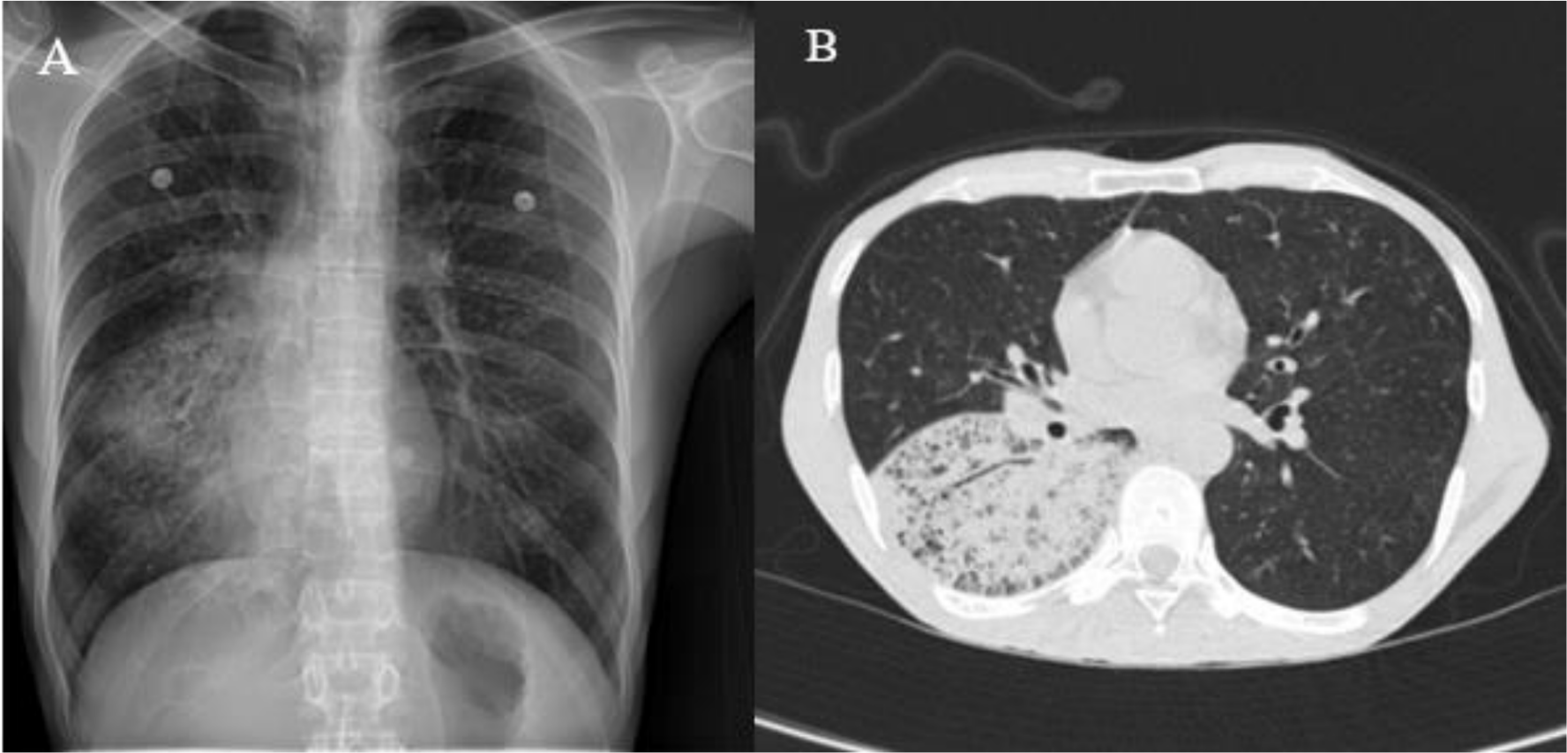
(**A**): Chest X-ray revealed infiltration in the right lung. (**B**): Lung HRCT scan revealed “crazy-paving” appearance and interstitial infiltration and interlobular septal thickening localized in the superior segment of the right lower lobe

**Fig. 2:**
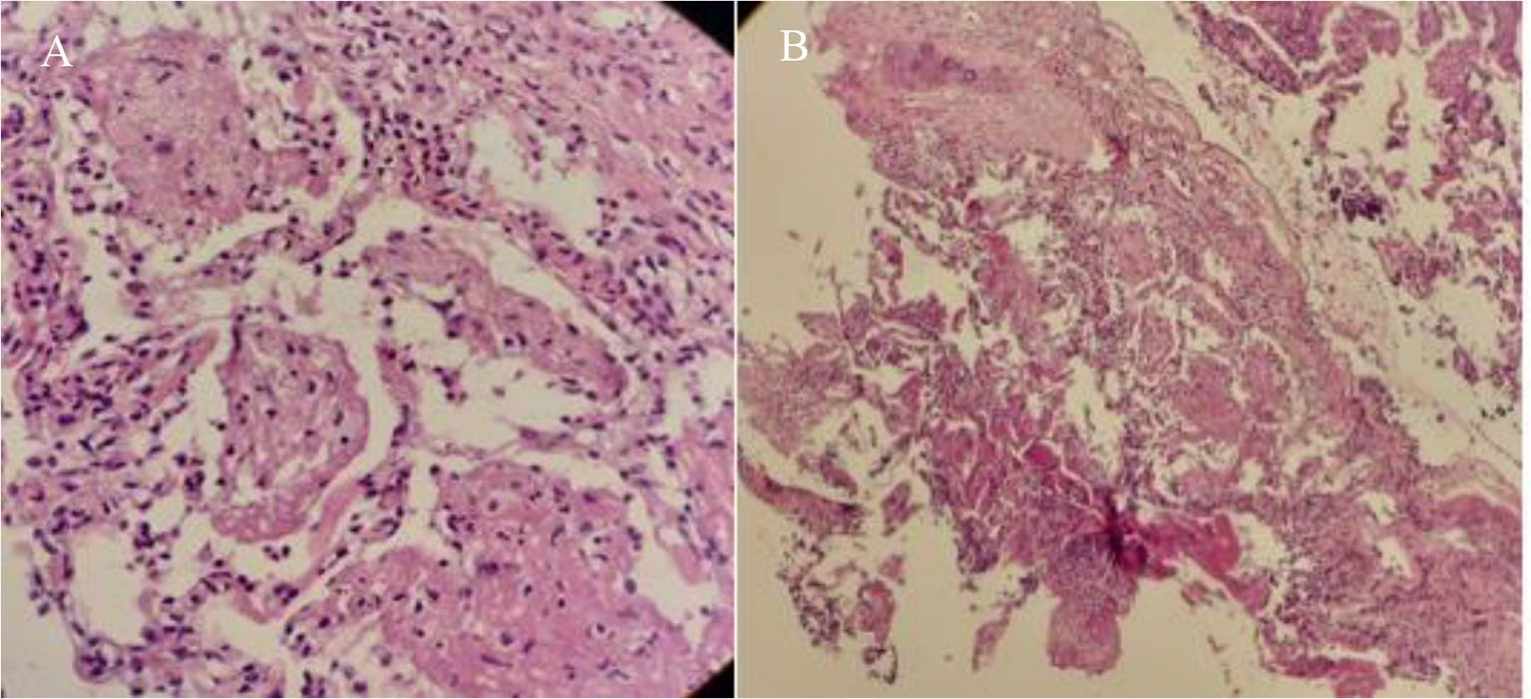
Histologic sections of the lung biopsy showed some destructed alveolar spaces with acute and chronic inflammation and some intra-alveolar foamy exudate which are suggestive of pneumocystis jirovecii pneumonia, (**A**): High power field (magnification of ×400). (**B**): Low power field (magnification of ×100)

**Fig. 3:**
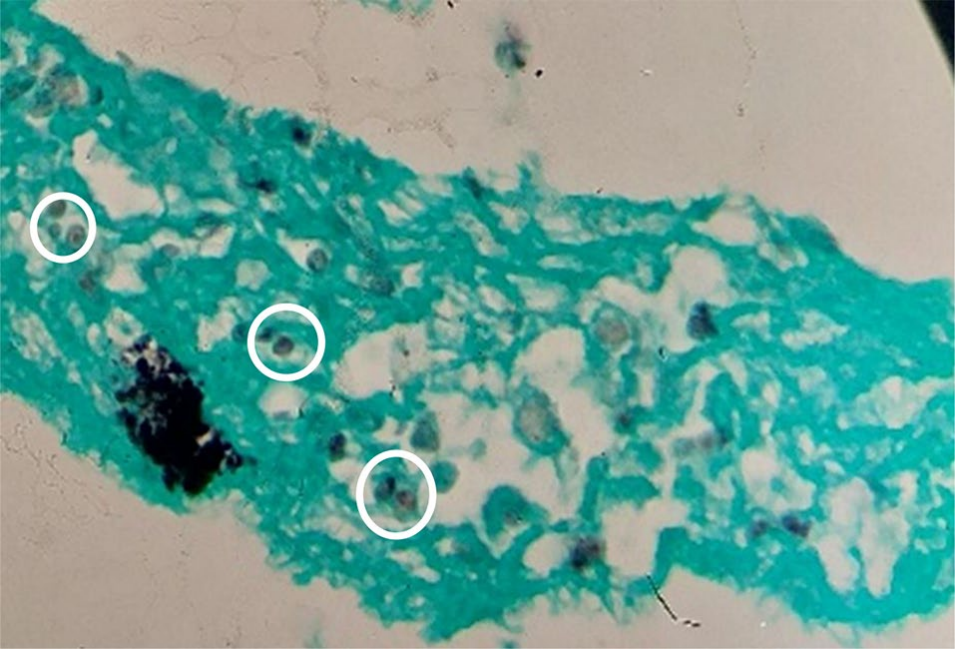
Multiple round microorganisms were visualized in hematoxylin and eosin (H&E) staining with methenamine silver (white circles), indicating Pneumocystis jirovecii (magnification of ×1000)

Radiographic investigations revealed interstitial infiltration in the right lower lobe, demonstrated in Fig. 1. Salmetrol/fluticasone (50/250 µg) was administered at 2 puffs every 12 h. Antibiotic therapy (ampicillin/sulbactam, 3 g every 6 h) was also initiated, and fiberoptic bronchoscopy was performed due to localized infiltration. Bronchoscopy revealed a normal tracheobronchial tree, so a transbronchial lung biopsy was performed from the superior segment of the right lower lobe bronchus. BAL fluid was evaluated by cytology, acid-fast stain, Mycobacterium Tuberculosis PCR, and conventional culture. The lung tissue biopsy and staining confirmed the diagnosis of PJP in the absence of malignancy (Figs. 2 and 3).

**Fig. 4:**
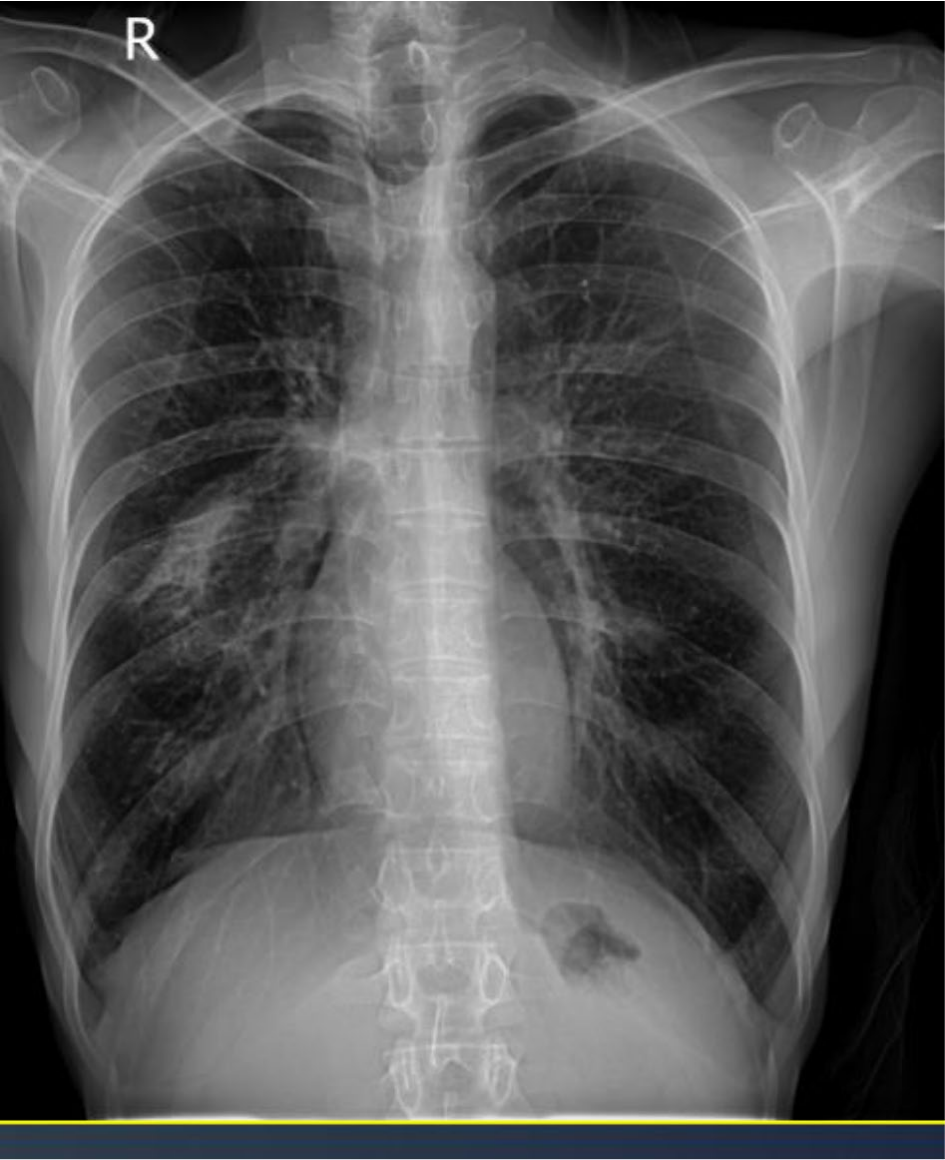
Follow-up chest X-ray showed significant improvement after 2 months of treatment, but the infiltration hadn’t been completely resolved

Trimethoprim-sulfamethoxazole was started orally at a dose of 20 mg/kg trimethoprim every 6 h, and the patient was followed on an outpatient basis after 6 days of hospitalization. Due to the relief of hypoxemia with bronchodilators and a short daily course of 30 mg prednisolone for COPD exacerbation management for 5 days, corticosteroids were not prescribed for PJP treatment. The patient was evaluated three weeks later; surprisingly, he gained weight, with complete resolution of cough, dyspnea and anorexia. The patient recovered from her cytopenia, and her serum immunoglobulin levels were also completely normal. HIV antigen and antibody were rechecked and reported to be negative for the second time in the reference laboratory. A chest X-ray was taken about 2 months after the end of treatment. There was evidence of radiological improvement, but a small amount of infiltration remained (Fig. 4), so a lung high-resolution computed tomography (HRCT) scan was requested. The patient refused to undergo a lung HRCT scan and continued his follow-up visits.

## Discussion and conclusions

Pneumocystis jirovecii pneumonia (PJP), formerly known as Pneumocystis carini, is an opportunistic infection affecting immunocompromised patients, especially HIV patients [4, 5]. HIV infection is the most common risk factor for PJP, but recently, cases of PJP in non-HIV patients have increased because of the wide use of immunosuppressive medications to treat malignancies, autoimmune diseases, and patients who underwent solid organ or hematologic stem cell transplantation [4, 6–9]. Some studies have shown that cigarette smoking is a risk factor for PJP in HIV-infected patients [10–13]. Studies have also shown that severe protein-calorie malnutrition is associated with PJP even in the absence of HIV infection [14–17], and a case of anorexia nervosa with severe respiratory failure was reported as PJP in a previous study [18]. Although the exact mechanism of immune system dysfunction has not been fully understood, vitamins, minerals and other micronutrient deficiency may be associated with immune system impairment. For instance, a sufficient vitamin A level is necessary to maintain a high CD4 count and can play an important role in cellular immunity, alongside zinc. Amino acids and fatty acids are also important substrates for various aspects of immune system. Therefore, malnutrition may lead to impaired immune function, an increased risk of developing infections and higher mortality from various infectious diseases [19–22]. Another possible risk factor for fungal infection in this patient is prolonged use of opium, which can significantly affect the immune system. Opium use has been associated with a decreased levels of cytokines such as interferon-gamma, interferon-alpha, IL-4, and IL-6, along with an increase in plasma levels of TGF-β, according to some studies [23, 24]. On the other hand, some studies have reported an increase in interferon-gamma plasma levels among individuals with opium addiction [25, 26]. Opium use can also increase lymphocyte counts which contrasts with our patient’s findings, so his cytopenia may be due to the pneumocystis jiroveci infection itself [27].

The clinical manifestations of PJP include nonspecific constitutional symptoms, fever, dry cough and severe progressive dyspnea. Physical examination findings may be normal or can reveal mild crackles on lung auscultation [7, 28, 29]. Chest X-rays may be completely normal even in the presence of infection and hypoxemia or can show localized infiltrations. Lung HRCT is more sensitive than plain radiography and can reveal a wide range of abnormalities, such as central interstitial infiltration and ground glass opacity with upper lobe predilection, which are the typical radiological presentations of PJP. Radiological findings may demonstrate diffuse ground glass opacities with a crazy-paving appearance, cystic changes and pneumothorax as the disease progresses. A lung HRCT scan of PJP patients may also show localized consolidation, which is more prevalent in non-HIV patients due to the immune response to infection [30–32]. Microbiologic diagnosis of PJP is based on direct visualization of microorganisms or PJP PCR in BAL fluid, induced sputum or sputum of suspected patients and direct detection of microorganisms in lung tissue [1, 3].

The patient described in this paper was a man with opium dependence, a heavy cigarette smoker (at least 60 pack-year) and non-HIV-infected man who seemed malnourished with BMI of 16.32 (weight = 50Kg, height = 175 cm). He had significant temporal muscle wasting, transient leukopenia and a localized site of infiltration with a crazy-paving appearance in the superior segment of the right lower lobe, which are not common findings as diffuse ground glass opacities. According to these primary findings, the first differential diagnosis was lepidic growth of lung adenocarcinoma, so fiberoptic bronchoscopy and transbronchial lung biopsy were performed. Surprisingly, pathology revealed PJP microorganisms within the lung tissue, so proper medication was started, and the patient’s symptoms, including dyspnea and cough, anorexia and leukopenia, recovered. It remains unknown whether the patient’s anorexia and malnutrition predisposed him to the infection or were complications of the disease.

In conclusion, we report a case of Pneumocystis jirovecii pneumonia (PJP), which is an opportunistic infection that mostly affects patients with HIV infection or other immunosuppressive conditions. Malnutrition among non-HIV patients can be a risk factor for PJP, and this group of patients may present with lobar pneumonia rather than diffuse ground glass opacities, which are more common presentations.

### Limitations

Due to the high cost and lack of insurance coverage of flow cytometry and other quantitative and qualitative immune system tests in our country, specific lymphocyte subclasses analysis couldn’t be conducted. Therefore, no further para clinical work-up was performed as the patient achieved significant clinical improvement and cytopenia resolution.

## Data Availability

Data is provided within the manuscript. Further details or additional data are available upon request.

## Abbreviations

PJP: Pneumocystis jirovecii pneumonia
HIV: Human immunodeficiency virus
HRCT: High-resolution computed tomography
BAL: Bronchoalveolar lavage
PCR: Polymerase chain reaction
H&E: Hematoxylin and eosin
COPD: Chronic obstructive pulmonary disease
